# Dissecting the pathogenic effects of ambient air pollution exposure and its blood DNA methylation markers on cardiovascular disease risk

**DOI:** 10.1186/s13148-025-02016-6

**Published:** 2025-12-19

**Authors:** Weipeng Li, Weiya Kong, Chaonan Shen, Huimin Fan, Yunli Shen, Yuzhen Zhang, Liang Zheng

**Affiliations:** 1 Research Center for Translational Medicine, Shanghai East Hospital, Tongji University School of Medicine, Shanghai, China; 2 State Key Laboratory of Cardiovascular Diseases and Medical Innovation Center, School of Medicine, Shanghai East Hospital, Tongji University, Shanghai, China; 3 Department of Epidemiology and Public Health, Tongji University School of Medicine, Shanghai, China; 4 Institute of Integrated Traditional Chinese and Western Medicine for Cardiovascular Chronic Diseases, Tongji University School of Medicine, Shanghai, 200120 China

**Keywords:** Ambient air pollution, Cardiovascular disease, Mendelian randomization, DNA methylation, Cohort study

## Abstract

**Background:**

Cardiovascular diseases (CVD) are influenced by a number of factors, including environmental and genetic components. By linking prospective cohort studies with epigenetics and CVD outcomes, it may be possible to gain insight into the complex mechanisms underlying CVD. This study aims to evaluate the impact of air pollution on CVD and investigate whether DNA methylation (DNAm) mediates the association between air pollution and CVD.

**Methods:**

In the prospective cohort study, the relationship between air pollutants and CVD incidence was analyzed using Cox regression. Dose–response was assessed by the restricted cubic spline model, and multiple pollutants’ impact was evaluated by the weighted quantile sum model. The link between genetically predicted DNAm sites related to air pollutants and CVD risk was explored through epigenetic Mendelian randomization (MR), with further evidence provided by gene colocalization analysis.

**Results:**

For every 10 μg/m^3^ increase, particulate matter with diameters less than 2.5 μm (PM_2.5_), particulate matter with diameter less than 10 μm (PM_10_), nitrogen dioxide (NO_2_), and sulfur dioxide (SO_2_) increased the risk of CVD by 6.2%, 4.4%, 9.3%, and 6.1%, respectively, with all showing a linear association. Of the four air pollutants, PM_10_ and PM_2.5_ were identified as the most significant contributors to the CVD risk, accounting for 61% and 20%, respectively. Genetically predicted methylation at the PM_2.5_-related CpG site cg01065697 was linked to a higher risk of myocardial infarction (MI) and coronary heart disease (CHD), the NO_2_-related CpG site cg07091220 was associated with increased MI risk, the NO_2_-related sites cg15474579, cg16348358, and cg19869422 were linked to a higher risk of heart failure (HF).

**Conclusion:**

Our study confirms a significant association between air pollution, DNAm and CVD risk, and provides new insights into the pathogenic effects of air pollution on CVD.

**Supplementary Information:**

The online version contains supplementary material available at 10.1186/s13148-025-02016-6.

## Introduction

Cardiovascular diseases (CVD), including myocardial infarction (MI), coronary heart disease (CHD), angina, and heart failure (HF), continue to increase globally, representing a significant cause of mortality and disability worldwide [1]. In China, 40% of deaths can be attributed to CVD [2]. The etiology of CVD is multifaceted, influenced by various factors such as lifestyle, genetics, and the environment [3–7]. Notably, due to accelerated global industrialization, air pollutants including particulate matter with diameters less than 2.5 μm (PM_2.5_), particulate matter with diameter less than 10 μm (PM_10_), nitrogen dioxide (NO_2_), and sulfur dioxide (SO_2_) have emerged as a major public health hazard and have been found to be associated with an elevated risk of various diseases, including Parkinson’s disease, sarcopenia, and autoimmune disorders [8–10].

Although many epidemiological studies have examined the association between air pollution and CVD risk [11, 12], several key knowledge gaps remain. First, while prior research has examined the effects of individual air pollutants, investigations into the cardiovascular impacts of mixed pollutant exposures remain insufficient [13, 14]. Furthermore, although air pollution has been shown to induce systemic inflammation and oxidative stress [15, 16], the underlying biological mechanisms linking air pollution to CVD are not yet fully elucidated [17]. Emerging evidence suggests that DNA methylation (DNAm), a common epigenetic modification responsive to environmental exposures, may mediate the effects of air pollution on cardiovascular health [18–21]. Epigenome-wide association studies (EWAS) have demonstrated that air pollutants can alter DNAm patterns in blood and tissue samples [22–24], and accumulating evidence supports the involvement of DNAm in the pathogenesis of CVD.

To address the above research gaps, the present study adopts an integrative multi-step approach. First, we examined the association between long-term exposure to PM_2.5_, PM_10_, NO_2_, and SO_2_ (both single and mixed) and incident CVD using data from a national prospective cohort. Following this, we identified CpG sites associated with air pollution using publicly available EWAS data and evaluated their potential causal effects on four types of CVD (MI, CHD, angina, HF) via two-sample Mendelian randomization (MR) analysis. Finally, we conducted colocalization analysis to verify whether the DNAm–CVD associations shared common causal variants, thereby providing more robust mechanistic insights into how air pollution may contribute to CVD development at the molecular level.

## Methods

### Study design

**Fig. 1:**
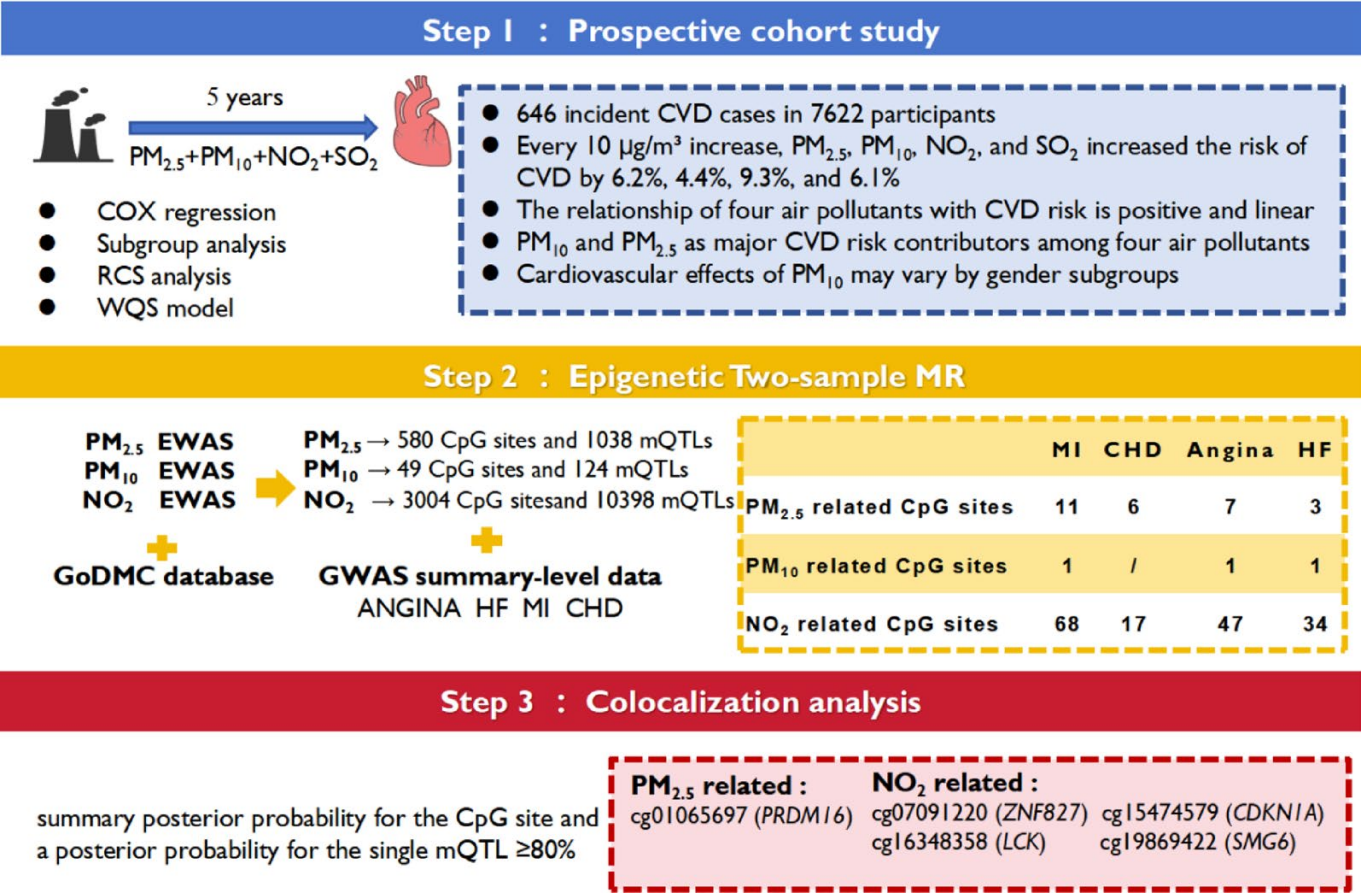
Schematic diagram of the study design

Figure 1 shows the study design overview. First, we investigated the association between multiple air pollutants and the risk of CVD incidence in a large five-year prospective cohort study and performed subgroup analyses to assess the effects of air pollutants on CVD incidence in specific subgroups. Restricted cubic spline (RCS) was used to further investigate the dose–response relationship between air pollutants and CVD incidence. Weighted quantile sum (WQS) regression model was also used to simulate the effects of mixed exposures to several air pollutants on CVD. Second, to explore the underlying mechanisms of the air pollutant-CVD association, we investigated the relationship between blood DNAm levels associated with air pollutants and multiple CVD using two-sample MR analyses. Third, we performed colocalization analyses of statistically significant CpG sites identified in epigenetic MR analyses to obtain more specific and robust evidence.

### Prospective cohort study

#### Study population

The data pertaining to the prospective cohort study were sourced from the China Health and Retirement Longitudinal Study (CHARLS), a national cohort study of Chinese adults aged 45 years and older. The study employed a multistage stratified probability proportional size sampling strategy to recruit participants from urban and rural areas across 28 provinces and 150 counties or districts in China. The CHARLS study is conducted in accordance with the principles set forth in the Declaration of Helsinki. The CHARLS study was approved by the Institutional Review Board of Peking University (IRB00001052-11015). The design and cohort profile of the CHARLS study have been previously described in detail in other article [25]. In this study, participants who were interviewed in 2015 were designated as the baseline cohort and subsequently followed up in 2018 and 2020. Figure S1 illustrates the inclusion and exclusion criteria for this study. The initial data extraction process yielded a sample of 17,806 participants who did not have CVD in 2015. Then, 330 participants with missing air pollution data, 327 participants with missing age data, and 3,960 participants with missing or excessive body mass index (BMI) values were subsequently excluded. Furthermore, 1,186, 4,588, and 1,690 participants with incomplete data on the three key confounding variables of residence, smoking status, and alcohol consumption, respectively, were excluded from the study. Ultimately, 7,622 participants were included in the final analyses.

### Assessment of air pollutants

The data on air pollution were obtained by collating the monthly National Reports on Urban Air Quality Conditions published by the China National Environmental Monitoring Centre (CNEMC) from 2013 to 2015. All pollutant concentrations were measured in accordance with the Chinese Ambient Air Quality Standards (GB 3095–2012), as described in previous studies [26]. The arithmetic mean of pollutant concentrations measured at all monitoring stations within each city was used to represent the regional average concentration in the built-up urban area. To validate this value, it was compared against estimates derived from measured or simulated data at densely distributed grid points (each no larger than 2 km × 2 km), with the relative error between the two values required to be within 10%. Monthly concentrations were calculated by averaging daily values, requiring at least 21 valid daily measurements to constitute a valid monthly mean. The three-year average exposure concentrations of PM₂.₅, PM₁₀, NO₂, and SO₂ were then calculated at the city level and assigned to each participant based on their residential address code. Accordingly, all individuals residing in the same city were assigned the same exposure value for each pollutant.

### Assessment of incidence of CVD

The principal objective of this study was to ascertain the incidence of CVD during the follow-up period. In accordance with the methodology employed in previous studies of a similar nature, we collated data pertaining to previous diagnoses of CVD through the use of a standardised question: “*Have you ever been diagnosed by a medical practitioner with heart attack, coronary heart disease, angina, congestive heart failure, or other heart problems?*” [27]. The CHARLS study team implemented rigorous quality control measures for data collection and validation, with the aim of ensuring the reliability of the data [25].

### Assessment of covariates

BMI was categorized as < 24 kg/m^2^ or ≥ 24 kg/m^2^, based on the Chinese Guidelines for Prevention and Control of Overweight and Obesity in Adults, which define a BMI of 24 kg/m^2^ or higher as overweight [28]. Smoking status was classified into three categories: never, former, and current, based on participants’ self-reported history of smoking and whether they had quit smoking at the time of the baseline assessment [29]. Similarly, drinking status was categorized as never, former, or current, according to both past and current alcohol consumption and drinking frequency [29]. Those participants who reported a history of hypertension or were undergoing treatment for hypertension, as well as those with an systolic blood pressure (SBP) of 130 mmHg or higher or a diastolic blood pressure (DBP) of 80 mmHg or higher at baseline, were defined as having hypertension [30]. Individuals who reported a history of diabetes or were undergoing treatment for diabetes, as well as individuals with a fasting blood glucose (FBG) level of 7.0 mmol/L (126 mg/dL) or above at the baseline assessment were considered to have diabetes [31, 32]. Dyslipidemia was defined as self-reported use of cholesterol-lowering medication or physician-diagnosed hyperlipidaemia, as well as total cholesterol levels exceeding 5.18 mmol/L, low-density lipoprotein (LDL) levels exceeding 3.37 mmol/L, and high-density lipoprotein (HDL) levels below 1.04 mmol/L (in males) or below 1.30 mmol/L (in females) [27]. The residential address of each participant was ascertained by means of a direct question, inquiring as to whether the address was located in an urban or rural setting.

### Statistical analysis

The Kolmogorov–Smirnov normality test was employed for continuous variables, while for those that did not conform to a normal distribution, the median and interquartile range were utilised for statistical description. The Kruskal–Wallis H-test was then applied to assess the existence of differences between groups. Categorical variables were presented as frequencies and percentages, and differences between groups were assessed using the chi-squared test. Four Cox regression models were employed to prospectively analyse the relationship between multiple air pollutants and the five-year incidence of CVD. Model I: crude model; Model II: adjusted for age, gender, and BMI; Model III: adjusted for age, gender, BMI, smoking status, drinking status, and residence; Model IV: adjusted for age, gender, BMI, smoking status, drinking status, residence, hypertension, diabetes, and dyslipidemia. To examine the relationship between air pollution and the incidence of CVD across different demographic characteristics, subgroup analyses were conducted based on different age groups (< 60 years vs. ≥ 60 years), gender, BMI (< 24 kg/m^2^ vs. ≥ 24 kg/m^2^), smoking status (never, former, current), and drinking status (never, former, current). Furthermore, RCS regression analyses were conducted to investigate the dose–response relationship between air pollution and CVD incidence. Subsequently, WQS model was employed to simulate real-life mixed exposures to multiple air pollutants, and the relative importance of the effects of individual air pollutants on CVD was assessed. The model was fitted to the complete data by performing 1,000 iterations of bootstrap in both the training set (40%) and validation set (60%) [33].

### Two-sample epigenetic Mendelian randomization

#### mQTL for air pollution-related DNA methylation

Firstly, blood DNAm associated with PM_2.5_, PM_10_, and NO_2_ exposure was obtained from three previous studies, comprising 2956, 365, and 2410 European participants, respectively. DNAm was measured by the Illumina Infinium HumanMethylation450 (HM450) BeadChip array. The relationship between air pollutants and blood DNAm was adjusted for age, gender, BMI, smoking status, alcohol intake, batch effect, and applicable leukocyte type using Illumina HumanMethylation450 (HM 450) BeadChip array measurements. In total, 1,829, 286, and 4,980 CpG sites were found to be associated with PM_10_, PM_10_, and NO_2_ exposure, respectively. Statistical significance was determined at an epigenomic level using the False Discovery Rate (FDR) correction method proposed by Benjamini and Hochberg, with FDR-adjusted P values less than 0.05 considered significant [34].

Subsequently, CpG site-associated methylation quantitative trait sites (mQTL) were identified from the Genetics of DNAm Consortium (GoDMC) database, which contains genetic and methylation data from over 30,000 individuals [35]. Significant cis mQTL (P < 1 × 10⁻⁸) located within 1 MB of the respective CpG site were identified through screening, and linkage disequilibrium (LD) was additionally applied to select independent instrumental variables for smoking-related DNAm (r^2^ < 0.01). The *F*-values were calculated for all mQTL and instrumental variables. Those with *F*-values greater than 10 were then screened for reliability [36]. The instrumental variables that have been identified as meeting all of the aforementioned screening conditions will be employed as genetic proxies for the DNAm induced by air pollution. In the assessment of the genetically predicted causal effects of DNAm at air pollution-related CpG sites on multiple CVD diseases, each air pollution-associated CpG site was considered as an independent exposure. This approach aligns with the use of the proxy mQTLs extracted from the GoDMC database as genetic tools.

### GWAS summary-level data of CVD

GWAS summary statistics for MI,CHD,ANGINA,HF are from the latest FinnGen consortium, which is a large, high-quality GWAS database. Strict quality control measures were implemented during the data collection process, resulting in the exclusion of individuals with ambiguous gender, high genotype missingness (> 5%), excess heterozygosity (± 4 SD), and non-Finnish ancestry. In the variant-wise quality control steps, variants with high missingness (> 2%), low Hardy–Weinberg equilibrium (HWE) *P*-value (< 1e-6), and low minor allele count (MAC < 3) were excluded [37].Table S1 shows the detailed sources of GWAS data on CVD.

### Two-sample MR

Two-sample MR analyses were conducted to elucidate the underlying biology by exploring the causal impact of air pollution-associated DNAm on the risk of CVD prevalence. Wald ratios were employed as the primary analytical tool for CpG site with a single mQTL, while inverse variance weighted (IVW) was utilised as the primary analytical tool for CpG site with a minimum of two mQTLs [38, 39]. FDR-adjusted P values < 0.05 were applied to determine statistical significance in multiplexed assays. Additionally, The heterogeneity of mQTLs and multiplicity of associations were evaluated using Cochran’s Q-statistic and the MR-Egger intercept test, respectively [40].

#### Colocalization of mQTLs and CVD GWAS signals

We extracted mQTL proxies for methylation at each CpG site from GoDMC. This was done for CpG sites that were significantly associated with an elevated risk of developing any of the CVD (FDR < 0.05) and contained greater than or equal to 10 usable mQTLs. To investigate whether the observed association between common variants and CVD was driven by shared effects on CpG site methylation, we conducted colocalization analyses using the ‘coloc’ package in R software.

In the colocalization analysis, five hypotheses were tested using the posterior probability approach.

H0: The genetic variant in the colocalised region is not associated with either trait.

##### H1

The genetic variant in the colocalised region is associated with the first trait but not the second.

##### H2

The genetic variant in the colocalised region is associated with the second trait but not the first.

##### H3

The genetic variant in the colocalised region is associated with both traits, but not at the same locus.

##### H4

Genetic variation within the colocalised region is associated with both traits and at the same locus.

An a posteriori association probability of 80% or higher for a pooled effect of CpG site and a single effect of an mQTL is regarded as evidence of colocalization [41].

## Results

### Baseline characteristics of prospective cohort study

A total of 7,622 participants were enrolled in the study, and after a five-year follow-up period, 646 participants had developed a new case of CVD, resulting in a new onset rate of 8.48%. Table S2 illustrates the distribution of the detailed baseline characteristics.

### Air pollution and risk of CVD incidence

Table 1 illustrates the correlation between exposure to the four air pollutants and the subsequent risk of developing CVD. Four Cox regression models were constructed for the four air pollutants (PM_2.5_, PM_10_, SO_2_, and NO_2_), with continuous variables transformed into units of 10 and categorical variables divided into quartiles. In the fully adjusted model (Model IV), each 10 μg/m^3^ increase in PM₂.₅ was associated with a 6.2% increase in CVD risk (hazard ratio [HR] = 1.062, 95% CI: 1.024–1.102). For PM₁₀, SO₂, and NO₂, the respective increases were 4.4% (HR = 1.044, 95% CI: 1.023–1.065), 6.1% (HR = 1.061, 95% CI: 1.012–1.112), and 9.3% (HR = 1.093, 95% CI: 1.022–1.168).

**Table 1:** Association between air pollution and CVD incidence in the prospective cohort study

	Model I (HR, 95%CI)	P	Model II (HR, 95%CI)	P	Model III (HR, 95%CI)	P	Model IV (HR, 95%CI)	P
PM_2.5_								
Each 10 μg/m^3^ increase	1.077(1.039,1.117)	< 0.001	1.079 (1.041,1.119)	< 0.001	1.081 (1.042,1.121)	< 0.001	1.062 (1.024,1.102)	0.001
Q1	(Reference)		(Reference)		(Reference)		(Reference)	
Q2	1.047(0.831,1.318)	0.697	1.019 (0.809,1.283)	0.876	1.023 (0.812,1.289)	0.849	1.002 (0.795,1.263)	0.987
Q3	1.286(1.028,1.609)	0.028	1.269 (1.015,1.586)	0.037	1.267 (1.012,1.586)	0.039	1.231 (0.983,1.541)	0.070
Q4	1.416(1.137,1.765)	0.002	1.426 (1.144,1.777)	0.002	1.435 (1.151,1.789)	0.001	1.321 (1.058,1.648)	0.014
PM_10_								
Each 10 μg/m^3^ increase	1.050(1.029,1.071)	< 0.001	1.052 (1.032,1.073)	< 0.001	1.053 (1.033,1.074)	< 0.001	1.044 (1.023,1.065)	< 0.001
Q1	(Reference)		(Reference)		(Reference)		(Reference)	
Q2	1.214(0.960,1.536)	0.106	1.208 (0.955,1.528)	0.116	1.202 (0.949,1.501)	0.127	1.164 (0.919,1.474)	0.207
Q3	1.325(1.051,1.671)	0.017	1.329 (1.054,1.675)	0.016	1.325(1.051,1.671)	0.017	1.285(1.019,1.621)	0.034
Q4	1.680(1.344,2.100)	< 0.001	1.727 (1.381,2.159)	< 0.001	1.733(1.356,2.168)	< .001	1.583 (1.263,1.982)	< 0.001
NO_2_								
Each 10 μg/m^3^ increase	1.116(1.044,1.192)	0.001	1.120 (1.048,1.197)	< 0.001	1.121 (1.049,1.198)	< 0.001	1.093 (1.022, 1.168)	0.010
Q1	(Reference)		(Reference)		(Reference)		(Reference)	
Q2	1.092(0.867,1.374)	0.456	1.092 (0.867,1.375)	0.454	1.089 (0.865,1.372)	0.468	1.057 (0.839, 1.332)	0.640
Q3	1.366(1.094,1.704)	0.006	1.372 (1.099,1.712)	0.005	1.365 (1.093,1.704)	0.006	1.343 (1.075,1.677)	0.009
Q4	1.338(1.073,1.670)	0.010	1.350 (1.082,1.680)	0.008	1.354 (1.084,1.690)	0.007	1.240 (0.993,1.550)	0.058
SO_2_								
Each 10 μg/m^3^ increase	1.007(1.002,1.012)	0.003	1.078 (1.029,1.130)	0.002	1.080 (1.030,1.132)	0.001	1.061 (1.012,1.112)	0.014
Q1	(Reference)		(Reference)		(Reference)		(Reference)	
Q2	0.845(0.670,1.066)	0.155	0.847 (0.672,1.068)	0.161	0.846 (0.671,1.067)	0.159	0.860(0.681,1.084)	0.202
Q3	1.111(0.897,1.376)	0.334	1.111 (0.897,1.375)	0.336	1.114 (0.899,1.380)	0.324	1.098 (0.887,1.360)	0.391
Q4	1.204(0.975,1.488)	0.085	1.234 (0.998,1.524)	0.052	1.241 (1.004,1.534)	0.046	1.166 (0.943,1.443)	0.157

Model I: Crude model;

Model II: Adjusted for age, gender, BMI;

Model III: Adjusted for age, gender, BMI, smoking status, drinking status, residence;

Model IV: Adjusted for age, gender, BMI, smoking status, drinking status, residence, hypertension, diabetes, dyslipidemia

In the quartile-based analysis using the fully adjusted model, the HR were 1.321 (95% CI: 1.058–1.648) for the Q4 group compared with Q1 for PM_2.5_, 1.583 (95% CI: 1.263–1.982) for PM_10_, 1.240 (95% CI: 0.993–1.550) for NO_2_ and 1.166 (95% CI: 0.943–1.443) for SO_2_.

**Fig. 2:**
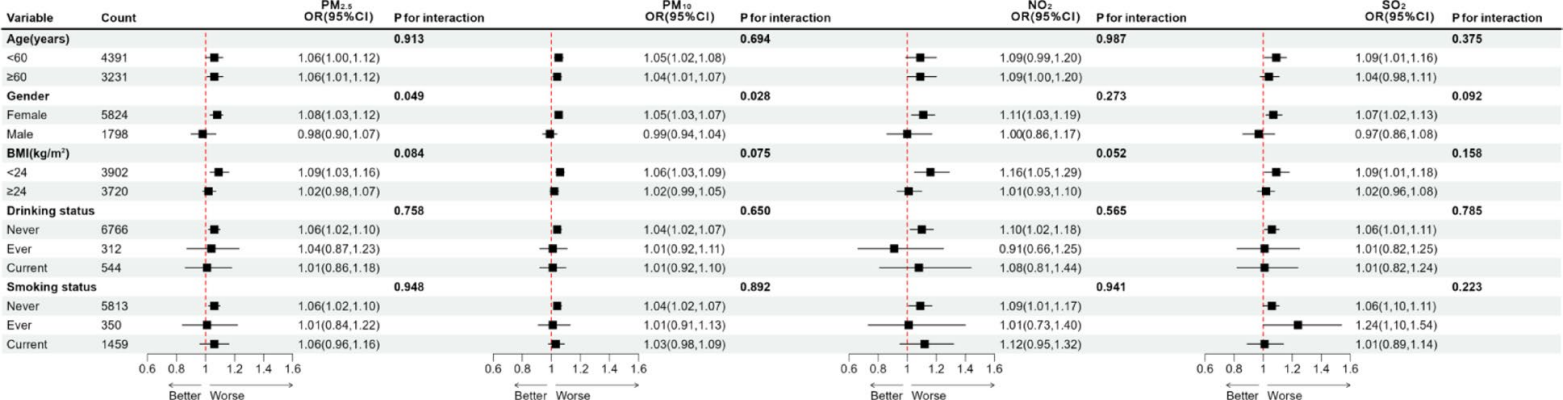
Subgroup analyses of the association between air pollution and CVD incidence. The model was adjusted for age, gender, BMI, smoking status, drinking status, residence, hypertension, diabetes, and dyslipidemia (excluding the variable used for subgroup stratification)

The results of the subgroup analyses indicated that the cardiovascular effects of PM_10_ differed significantly among gender subgroups, with a statistically significant interaction (P for interaction < 0.05). No significant differences were observed among the remaining air pollutant subgroups (Fig. 2).

**Fig. 3:**
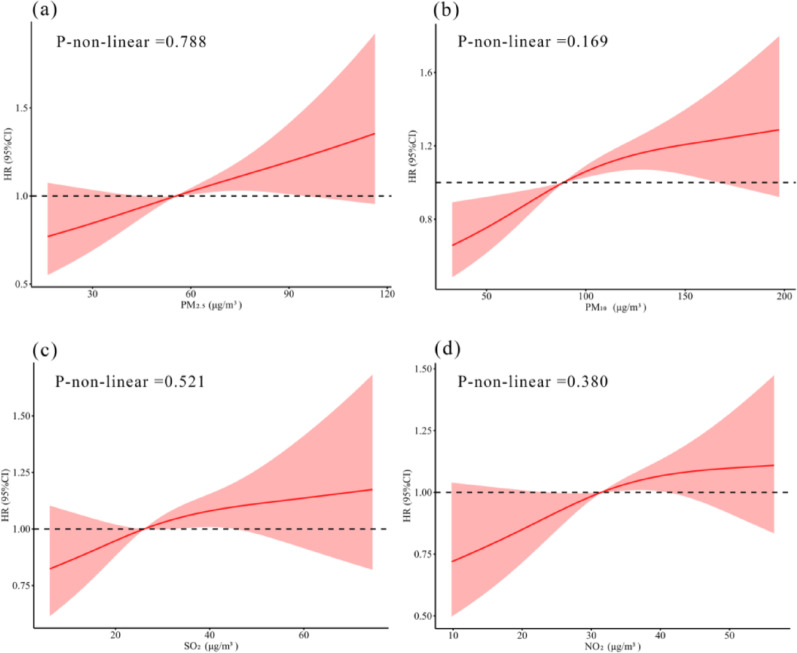
The RCS analysis between air pollution and CVD incidence. The model was adjusted for age, gender, BMI, smoking status, drinking status, residence, hypertension, diabetes, and dyslipidemia

The dose–response relationship between air pollutant exposure and the incidence of CVD are illustrated in Fig. 3. No nonlinear relationships were identified for any of the four air pollutants (P for nonlinear > 0.05).

**Fig. 4:**
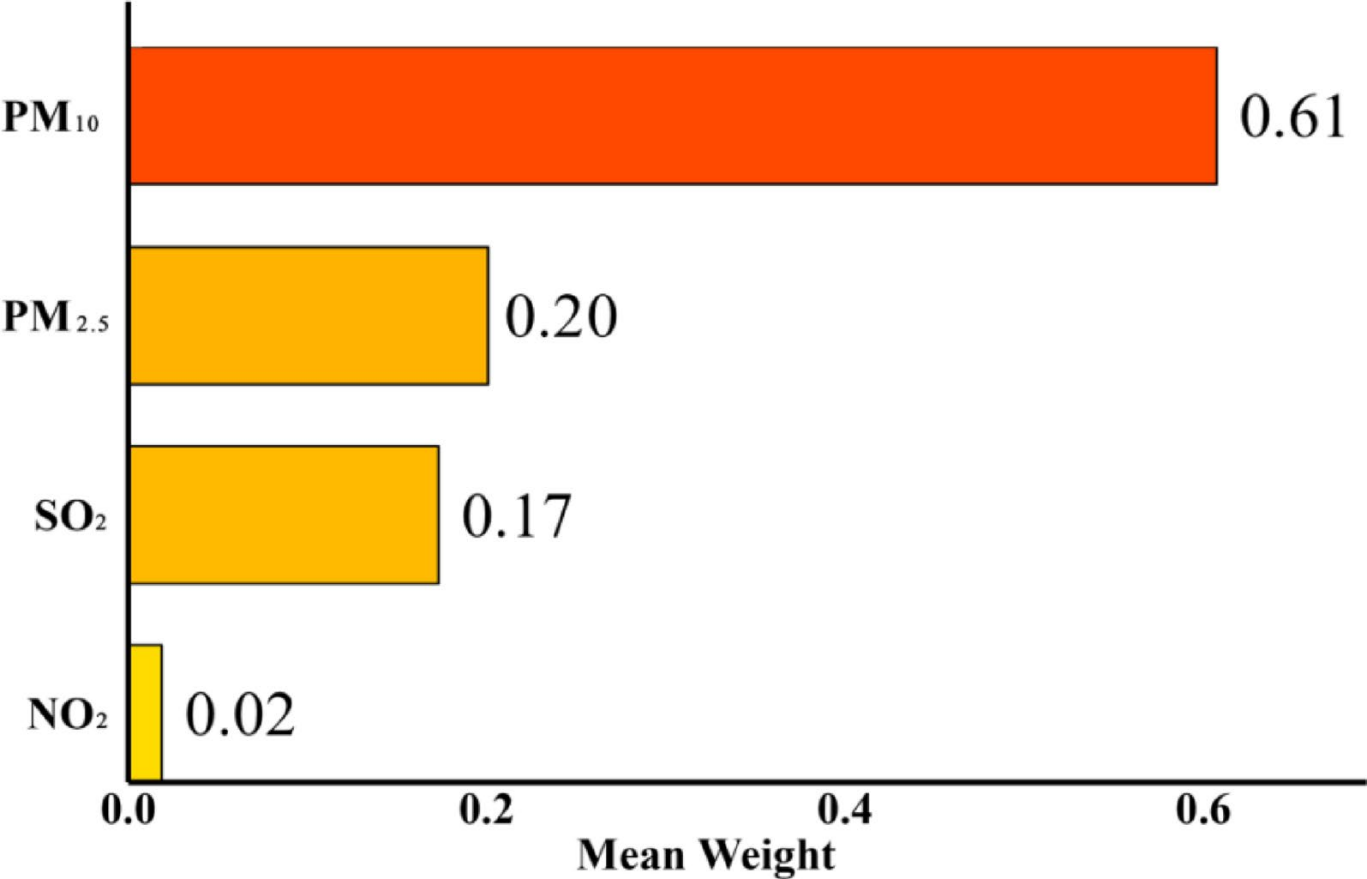
The relationship between mixed exposure to four air pollutants and cardiovascular disease risk based on the WQS model and their respective weights

The WQS regression model was fitted to the raw data of the four air pollutants (Fig. 4). The result revealed a statistically significant effect of mixed exposure to air pollution on the risk of developing CVD (P for mixture = 0.011), with an OR of 1.010 and a 95% CI of (1.002, 1.018). Furthermore, the result indicated that PM_10_ and PM_2.5_ contributed the most to the risk of CVD caused by co-exposure to the four air pollutants, with 61% and 20%, respectively.

### Air pollution-related DNA methylation and CVD risk

For the 1829, 284, and 4980 CpG sites previously identified as associated with PM_2.5_, PM_10_, and NO_2_, respectively, we applied a series of stringent screening criteria and extracted 580 CpG sites with 1038 usable cis-mQTLs as proxies for PM_2.5_-related methylation, 49 CpG sites with 124 available cis-mQTLs as proxies for PM_10_-related methylation, and 3004 CpG sites with 10,398 available cis-mQTLs as proxies for NO_2_-related methylation (Tables S3–S5).

Tables S6-S8 present the results of the Epigenetic Two-sample MR analysis for the methylation levels of all genetically predicted CpG site in relation to four CVD. We found that the methylation of 11, 6, 7, and 3 PM_2.5_-related CpG sites was significantly associated with an increased risk of MI, CHD, angina, and HF, respectively (FDR < 0.05). Notably, the CpG site cg01065697, located in the *PRDM16* gene, was significantly associated with the risk of MI, CHD, and angina simultaneously. Similarly, the methylation levels at cg14480594 in the ACTL7A gene, cg17387122 in the MICAL3 gene, and cg02787562 in the SUN1 gene, which are associated with PM_10_, were significantly positively correlated with the risk of MI, angina, and HF, respectively (FDR < 0.05). Moreover, methylation levels of 68, 17, 47, and 34 NO_2_-related CpG sites were significantly correlated with an increased risk of MI, CHD, angina, and HF, respectively (FDR < 0.05). Among these sites, cg04425005 in the *ZNF827* gene was simultaneously associated with increased risks of MI, CHD, and angina. Additionally, cg07091220, also located in the *ZNF827* gene, was significantly and positively correlated with the risks of all four CVD: MI, CHD, angina, and HF. The CpG site cg11407210 in the LTA gene was associated with increased risk of MI, CHD, and angina, while cg14437551 in the *CDKN1A* gene was linked to increased risk of MI, CHD, and angina. Furthermore, cg19869422 in the *SMG6* gene was associated with elevated risks of MI, CHD, and HF.

Cochran’s Q test was conducted for all CpG sites with more than two available cis-mQTLs, and no heterogeneity was observed (*P* > 0.05).

### Analysis of shared variants involved in determining specific CpG site methylation and CVD susceptibility

We conducted colocalization analysis for CpG sites that demonstrated a statistically significant association with increased CVD risk in the epigenetic MR analysis results (FDR < 0.05) and identified strong evidence of colocalization.

**Fig. 5:**
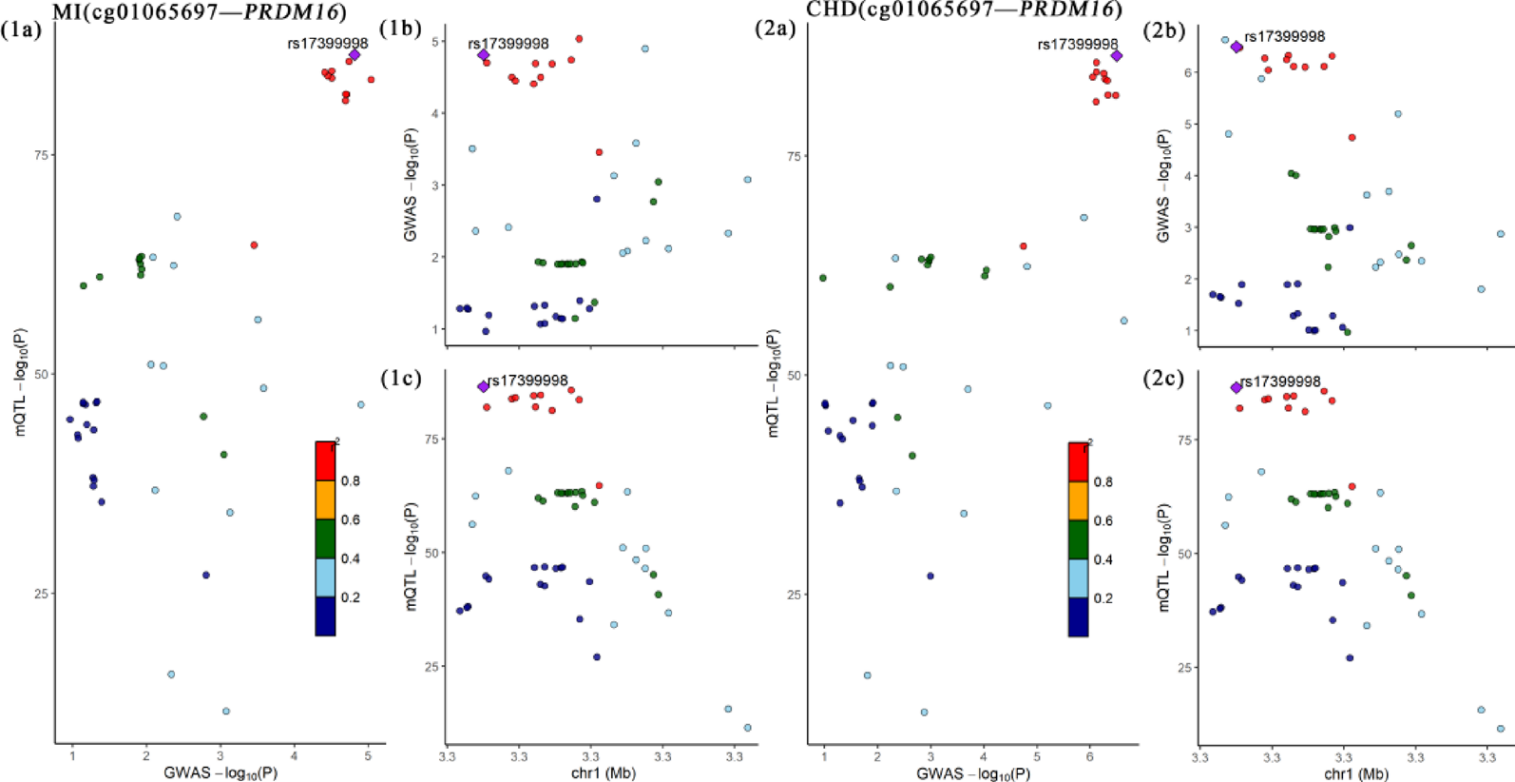
Regional plot of colocalization evidence of PM_2.5_ related-CpG site methylation and CVD (MI, CHD, HF) susceptibility. Each point represents a SNP. The results are presented in the form of “Disease (CpG site—Gene)” respectively. In figures **a** of each result, the − log10 (p) of SNPs from the corresponding GWAS and EWAS, as well as mQTL, are shown. In figures **b** and **c**, the x-axis represents the base positions of SNPs and mQTL, and the y-axis represents the − log10(p) of SNPs in the genome-wide association study and mQTL in the genome-wide DNAm analysis, respectively. SNPs that meet the colocalization threshold with an H4 posterior probability > 0.8 are highlighted in purple

For the CpG sites associated with PM_2.5_ (Fig. 5), we found that cg01065697, located in the *PRDM16* gene, had a posterior probability of 97.45% for sharing the same causal variant with MI and a posterior probability of 86.61% with the variant rs17399998. Additionally, cg01065697 shared a posterior probability of 86.61% for CHD with the same causal variant with a posterior probability of 99.21% and rs17399998 with a posterior probability of 99.20%.

**Fig. 6:**
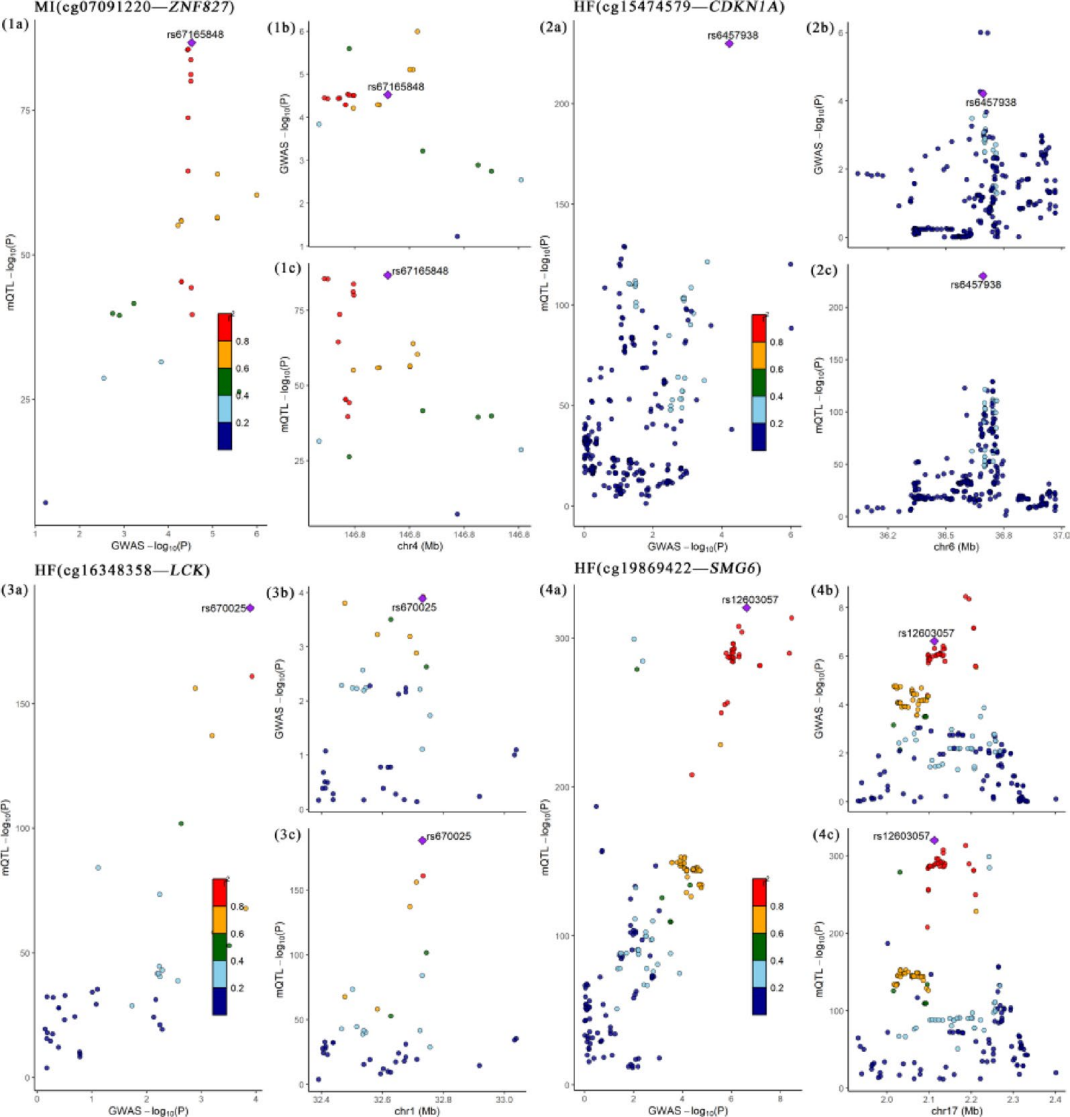
Regional plot of colocalization evidence of NO_2_ related-CpG site methylation and CVD (MI, HF) susceptibility. Each point represents a SNP. The results are presented in the form of “Disease (CpG site—Gene)” respectively. In figures **a** of each result, the − log10 (p) of SNPs from the corresponding GWAS and EWAS, as well as mQTL, are shown. In figures **b** and **c**, the x-axis represents the base positions of SNPs and mQTL, and the y-axis represents the − log10(p) of SNPs in the genome-wide association study and mQTL in the genome-wide DNAm analysis, respectively. SNPs that meet the colocalization threshold with an H4 posterior probability > 0.8 are highlighted in purple

For the CpG sites associated with NO_2_ (Fig. 6), we found that cg07091220 in the *ZNF827* gene shared the same causal variant with MI with a posterior probability of 91.48% and rs67165848 with a posterior probability of 89.88%. Furthermore, cg15474579 in the *CDKN1A* gene shared the same causal variant with a posterior probability of 81.40% and rs6457938 with a posterior probability of 100%. Additionally, cg16348358 in the *LCK* gene shared the same causal variant with HF with a posterior probability of 86.38% and rs670025 with a posterior probability of 100%, while cg19869422 in the *SMG6* gene shared the same causal variant with HF with a posterior probability of 89.00% and rs12603057 with a posterior probability of 99.99%.

Table 2 shows the results of the MR analysis of the CpG sites screened in the colocalization analysis with CVD. Notably, The genetically predicted methylation level of cg01065697 (located in the *PRDM16* gene and associated with PM_2.5_ exposure) is associated with an increased risk of MI and CHD, with MR estimates of 1.287 (95% CI = 1.162–1.425) and 1.258 (95% CI = 1.136– 1.394), respectively. For NO_2_, the genetically predicted methylation of cg07091220 (located in the *ZNF827* gene) is associated with an increased risk of MI, with an MR estimate of 1.243 (95% CI = 1.129–1.369). Furthermore, The genetically predicted methylation of cg15474579 (located in the *CDKN1A* gene), cg16348358 (located in the *LCK* gene), and cg19869422 (located in the *SMG6* gene) are all associated with an increased risk of HF, with MR estimates of 1.143 (95% CI = 1.115–1.173), 1.125 (95% CI = 1.051–1.205), and 1.103 (95% CI = 1.062–1.145), respectively.

**Table 2:** Two-sample Mendelian Randomization estimates of air pollution-related methylation on CVD risk

Exposure	CpG	Gene	Outcome	Method	n_mQTL	OR	Lower 95% CI	Upper 95% CI	P value	FDR
PM_2.5_	cg01065697	*PRDM16*	MI	Wald ratio	1	1.287	1.162	1.425	1.32E-06	1.19E-04
PM_2.5_	cg01065697	*PRDM16*	CHD	Wald ratio	1	1.258	1.136	1.394	1.04E-05	3.73E-03
NO_2_	cg07091220	*ZNF827*	MI	Wald ratio	1	1.243	1.129	1.369	9.03E-06	9.04E-04
NO_2_	cg15474579	*CDKN1A*	HF	IVW	8	1.143	1.115	1.173	4.22E-25	4.02E-22
NO_2_	cg16348358	*LCK*	HF	IVW	2	1.125	1.051	1.205	7.46E-04	3.15E-02
NO_2_	cg19869422	*SMG6*	HF	IVW	2	1.103	1.062	1.145	3.13E-07	2.71E-05

## Discussion

In the present study, we employed a comprehensive approach, comprising a large prospective cohort study, epigenetic MR analysis and genetic colocalization analyses. The findings of our study indicate that there is a significant positive linear association between exposure to air pollutants and the risk of developing CVD. Furthermore, our results demonstrate that exposure to a mixture of air pollutants is associated with an increased risk of CVD. It is noteworthy that elevated DNAm levels induced by air pollutants may, to some extent, mediate this association.

At the population level, studies have explored the association between air pollutants and CVD. However, these studies have mainly focused on fine particulate matter and have paid insufficient attention to other important air pollutants, such as NO₂; furthermore, the majority of studies have not adequately considered mixed exposures to a variety of air pollutants in real environments. In this study, we employed Cox regression modelling to verify the correlation between air pollutants and CVD events and further identified a significant linear relationship between air pollution and CVD risk through RCS modelling. Additionally, we utilised the WQS model analysis to examine the overall adverse effects of mixed exposures to multiple air pollutants on CVD risk and to establish the relative contribution of different air pollutants in the mixed exposures.

A meta-study demonstrated that ambient exposure to PM_2.5_ and PM_10_ was associated with elevated levels of circulating C-reactive protein (CRP), indicating that ambient exposure to PM_2.5_ and PM_10_ may contribute to a systemic inflammatory state [42]. In addition, exposure to fine particulate matter is highly associated with cardiovascular risk factors such as thrombosis and coagulation, oxidative stress, and atherosclerosis [11]. The results of an eight-year longitudinal study demonstrated that a 10 μg/m^3^ increase in NO₂ was associated with a 55.8% increase in overall CVD risk, with an HR of 1.56 (95% CI: 1.48–1.64). This finding aligns with the trend observed in our analysis [43]. It has been demonstrated that inhaled NO₂ induces cardiac dysfunction in male mice and that NO₂ exposure disrupts Ca^2^⁺ homeostasis, actin cytoskeleton reorganisation, myocardial contractility and vasodilation in male mice, as evidenced by transcriptional analysis results [44]. Furthermore, It has been demonstrated that SO₂ inhalation in mice elevates the risk of CVD by triggering inflammatory responses and injury through the activation of pro-inflammatory and pro-apoptotic genes in the heart and lungs [45].

The PM_2.5_-related CpG site cg01065697 was annotated to the gene *PRDM16*. It was demonstrated that *PRDM16* plays a role in maintaining the transcriptome properties of dense cardiomyocytes, acting to activate dense cardiomyopathy genes while repressing those associated with left ventricular trabecular cardiomyopathy. Furthermore, *PRDM16* has been shown to interact with left ventricle-rich transcription factors, enabling synergistic and specific regulation of left ventricular transcription. The knockdown of *PRDM16* in mouse cardiomyocytes by researchers resulted in the development of left ventricular dense incomplete cardiomyopathy, characterised by left ventricle-specific dilatation and dysfunction [46]. Furthermore, *PRDM16* has been identified as a key activator of beige adipocyte biogenesis, conferring protection against metabolic disorders [47]. This occurs through the formation of complexes with transcriptional and epigenetic factors [48]. The alterations and transcriptional dysregulatory effects of the systemic single-allele *Prdm16* gene inactivated *Prdm16*^csp1/wt^ mice affect the cardiac tissue, demonstrating that metabolic dysregulation is an early event in *PRDM16*-associated cardiology [49]. The present study identified a correlation between the methylation level of the *PRDM16* gene and an elevated risk of MI and CHD. This finding offers novel insights and evidence regarding the mechanism through which the *PRDM16* gene affects cardiac function.

Our study identified that the NO_2_-related CpG site cg07091220, located in *ZNF827*, is linked to an increased risk of MI. DNA damage is a major contributor to vascular aging [50], and previous research has established the crucial role of *ZNF827* in DNA damage repair and replication processes. The loss of *ZNF827* inhibits replication initiation and fork progression, leading to the accumulation of DNA damage [51]. Our findings suggest that NO_2_ exposure might increase the methylation level at cg07091220, thereby reducing *ZNF827* expression and consequently increasing MI risk. Additionally, we discovered that cg15474579, related to NO_2_ and located in the *CDKN1A* gene, is associated with an elevated risk of HF. The p21 protein encoded by the *CDKN1A* gene is a member of the CIP/KIP family [52] and is involved in cell cycle arrest, DNA repair, cell proliferation, and differentiation [53]. Recent studies indicate that p21 enhances the self-renewal of hematopoietic stem cells (HSCs) by co-repressing cKit expression with the transcriptional repressor ZBTB18, independent of its traditional cell cycle inhibitory function [54]. This potentially promotes cardiomyocyte proliferation and repair, thereby alleviating HF to some extent. Our results suggest that increased methylation at cg15474579 may reduce *CDKN1A* expression, thus increasing the risk of HF, offering a new perspective on the role of the *CDKN1A* gene in CVD mechanisms.

This study also revealed an association between methylation of cg16348358 at the *LCK* gene locus and an increased risk of HF. At present, there is a paucity of studies examining the direct effects of *LCK* on the cardiovascular system. The lymphocyte-specific protein tyrosine kinase encoded by the *LCK* gene plays a crucial role in the development and maturation of T cells [55]. Accordingly, we postulate that elevated levels of cg16348358 methylation may result in the down-regulation of *LCK* gene expression, which in turn affects the normal development and function of T-cells, leading to deficiencies in the immune response and thus the repair and responsiveness of cardiac tissues [56]. Another methylation site we found associated with an increased risk of HF is cg19869422, located in *SMG6*. Studies have shown that *SMG6*, as an important component of the NMD complex [57], facilitates the efficient degradation of mutant mRNA with premature stop codons through mechanisms such as endonucleolytic cleavage, preventing the accumulation of abnormal proteins [58, 59]. We speculate that this may, to some extent, reduce the likelihood of events detrimental to cardiomyocytes, such as cellular stress response, oxidative stress, and inflammation, thereby promoting the normal functioning of cardiomyocytes and reducing the risk of HF. In conclusion, the results of this study indicate that genes such as *ZNF827*, *CDKN1A*, *LCK*, and *SMG6* play a significant role in regulating cardiovascular health. Furthermore, they provide an epigenetic perspective that can inform further investigation of the relationship between NO_2_ and CVD.

The strength of this study lies in its comprehensive analytical approach, which included prospective cohort studies, epigenetic MR analysis and genetic colocalisation analyses. This enabled causal inferences to be made between air pollution, DNAm and CVD, and provided insights into how and why air pollution contributes to CVD. Nevertheless, the present study is not without limitations. Firstly, in the prospective cohort study, although we adjusted for some important confounders such as gender, smoking status, and drinking status, we were missing information such as family history of CVD, which is one of the most important factors influencing the development of CVD. Secondly, the exposure of each individual to air pollutants was estimated based on air pollutant monitoring data from their city of residence. This approach may have introduced misclassification bias. To minimise this bias, we calculated the average of three years of air pollutant data as each individual’s long-term exposure and included the variable indicating the participant’s place of residence (urban / rural). Thirdly, the air pollution-related DNAm data employed in this study was derived from a cross-sectional EWAS. Consequently, it was not possible to assess the long-term longitudinal effects of air pollution on DNAm. Therefore, further investigation is required through the utilisation of large longitudinal cohort studies in order to gain a deeper understanding of the relationship between air pollution-related DNAm and CVD. Fourth, the DNAm data in this study were at the pooled level, which precludes the possibility of forming a picture of the effects of air pollution-associated DNAm on CVD in populations with different specificities. Consequently, further in-depth analyses will be required when individual-level data become available. Fifth, the methylation sites identified in this study are genetic proxies, and thus functional experimental studies are needed to further validate their specific mechanisms of action on CVD.

## Conclusion

The findings of this study offer valuable insights into the potential causal role of air pollution in the aetiology of CVD. The present study has demonstrated a clear association between air pollutants (both single and mixed) and the risk and form of CVD in a large national cohort. Furthermore, we present compelling evidence that DNAm may be a crucial mechanism through which this association is mediated. Specifically, DNAm sites located at *PRDM16*, *ZNF827*, *CDKN1A*, *LCK*, and *SMG6* may serve as mediators of the causative effects of air pollutants on CVD pathogenesis. These findings offer promising avenues for investigating the pathogenesis of CVD and for developing new pharmacological and non-pharmacological interventions based on epigenetic mechanisms to prevent and treat CVD.

## Supplementary Information

Below is the link to the electronic supplementary material.

Supplementary Material 1

Supplementary Material 2

## Data Availability

The results of this study are presented in this article and its Supplementary files. The China Health and Retirement Longitudinal Study (CHARLS) dataset, which was utilized in our research, is publicly available. The air pollution data was sourced from the monthly national city air quality status report published by the China National Environmental Monitoring Centre (CNEMC) (https://www.cnemc.cn/). Genetic data was obtained from the FinnGen Consortium. Genetics of DNAm Consortium (GoDMC) and genome-wide DNAm analyses, which can be required from the published articles.
