# Supplementary material for: Dissecting the pathogenic effects of ambient air pollution exposure and its blood DNA methylation markers on cardiovascular disease risk
Source: Clin Epigenetics. 2025 Dec 19;17:205. doi: 10.1186/s13148-025-02016-6 (PMC12717718; doi:10.1186/s13148-025-02016-6)
Supplement: Supplementary file 1 — Supplementary Material 1 [file 13148_2025_2016_MOESM1_ESM.docx]

**Description of Additional Supplementary File 1**

**File Name: Supplementary Figure 1**

**Description: Flowchart of the prospective cohort study population.**

**File Name: Supplementary Table 1**

**Description: Data aources for GWAS on cardiovascular diseases.**

**File Name: Supplementary Table 2**

**Description: Baseline characteristics of the prospective cohort study population.**

**
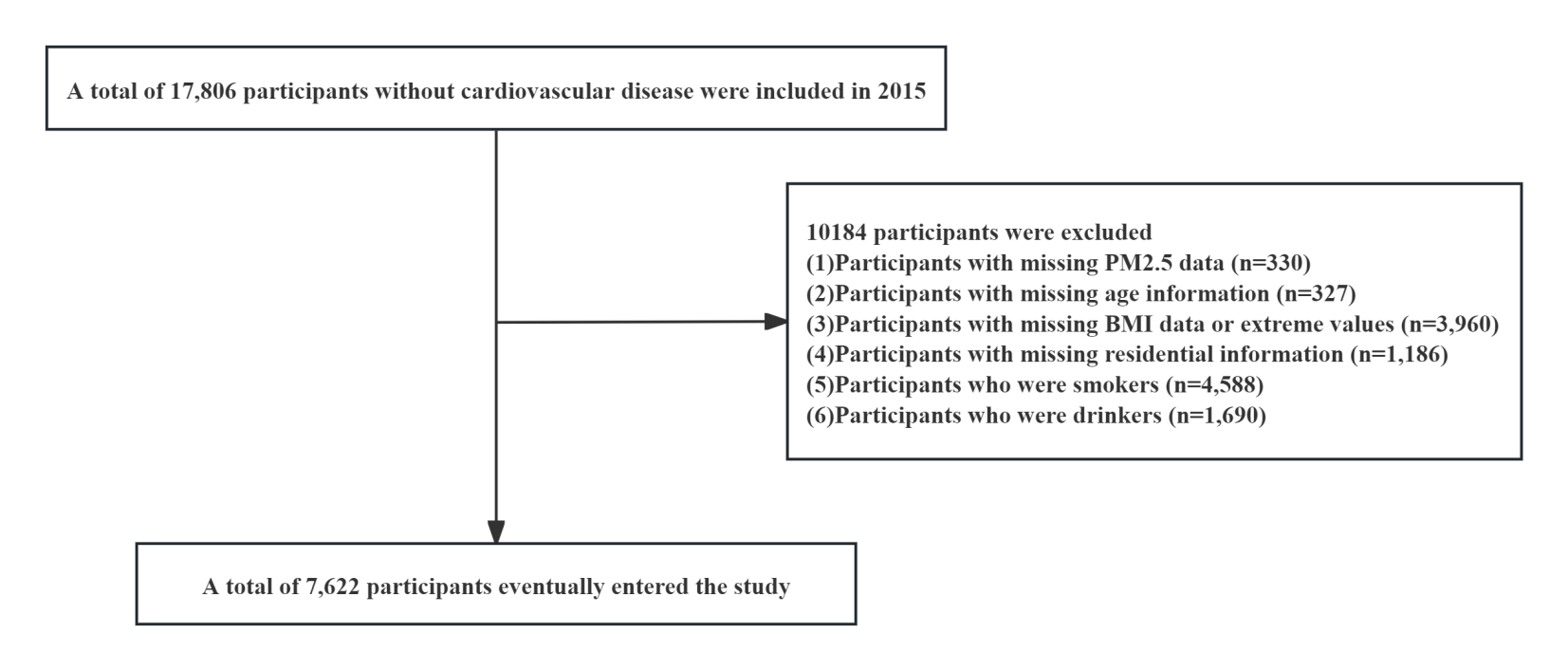
**

**Figure S1. Flowchart of the prospective cohort study population.**

**Table S1 .Data aources for GWAS on cardiovascular diseases.**

| **Trait** | **GWAS ID** | **Consortium** | **Sample size** |  | **Number of snps** | **Population** | **Case/Control** |
| --- | --- | --- | --- | --- | --- | --- | --- |
| ANGINA | finngen_R11_I9_ANGINA | FinnGen Consortium | 418385 | 0.0965 | 20093700 | European | 40366/378019 |
| CHD | finngen_R11_I9_CHD | FinnGen Consortium | 453733 | 0.1126 | 20094122 | European | 51098/402635 |
| HF | finngen_R11_I9_HEARTFAIL_ALLCAUSE | FinnGen Consortium | 452488 | 0.0723 | 20094104 | European | 32733/419755 |
| MI | finngen_R11_I9_MI_STRICT | FinnGen Consortium | 406565 | 0.0702 | 20093538 | European | 28546/378019 |

**Table S2.Baseline characteristics of the prospective cohort study population.**

| Variables | Overall (N = 7622) | non-CVD (N = 6976) | CVD (N= 646) | *P* |
| --- | --- | --- | --- | --- |
|  |  |  |  |  |
| Age, years | 57.42 (49.58, 65.25) | 56.92 (49.42, 65.00) | 61.29 (52.17, 67.75) | <0.001 |
| BMI, kg/m2 | 23.88 (21.58, 26.38) | 23.78 (21.51, 26.28) | 24.68 (22.51, 27.29) | <0.001 |
| Gender, n(%) |  |  |  | 0.018 |
| Famale | 5824 (76.41) | 5306 (76.06) | 518 (80.19) |  |
| Male | 1798 (23.59) | 1670 (23.94) | 128 (19.81) |  |
| Residence, n(%) |  |  |  | 0.954 |
| Rural | 1954 (25.64) | 1789 (25.65) | 165 (25.54) |  |
| Urban | 5668 (74.36) | 5187 (74.35) | 481 (74.46) |  |
| Smoke, n(%) |  |  |  | 0.418 |
| Never | 6766 (88.77) | 6191 (88.75) | 575 (89.01) |  |
| Former | 312 (4.09) | 281 (4.03) | 31 (4.80) |  |
| Current | 544 (7.14) | 504 (7.22) | 40 (6.19) |  |
| Drink, n(%) |  |  |  | 0.121 |
| Never | 5813 (76.27) | 5306 (76.06) | 507 (78.48) |  |
| Former | 350 (4.59) | 316 (4.53) | 34 (5.26) |  |
| Current | 1459 (19.14) | 1354 (19.41) | 105 (16.25) |  |
| Hypertension, n(%) | 3062 (40.17) | 2694 (38.62) | 368 (56.97) | <0.001 |
| Diabetes, n(%) | 879 (11.53) | 769 (11.02) | 110 (17.03) | <0.001 |
| Dyslipidemia, n(%) | 6230 (81.74) | 5656 (81.08) | 574 (88.85) | <0.001 |
| PM _2.5_, μg/m^3^ | 55.54 (41.31, 66.96) | 54.71 (41.01, 66.96) | 58.15 (43.12, 68.12) | <0.001 |
| PM _10_, μg/m^3^ | 88.62 (66.26, 113.36) | 87.38 (66.26, 113.24) | 93.21 (69.33, 125.86) | <0.001 |
| SO_2_, μg/m^3^ | 26.08 (18.79, 39.94) | 25.60 (18.79, 38.46) | 27.15 (18.79, 44.43) | 0.005 |
| NO_2_, μg/m^3^ | 31.44 (22.69, 40.16) | 31.38 (22.69, 40.16) | 32.33 (24.58, 42.50) | 0.001 |
